# Activity and abundance of methanotrophic bacteria in a northern mountainous gradient of wetlands

**DOI:** 10.1111/1758-2229.13137

**Published:** 2023-02-14

**Authors:** Sigmund Jensen, Henri M.P. Siljanen, Peter Dörsch

**Affiliations:** ^1^ Department of Biological Sciences University of Bergen Bergen Norway; ^2^ Department of Environmental and Biological Sciences University of Eastern Finland Kuopio Finland; ^3^ Norwegian University of Life Sciences Faculty for Environmental Sciences and Natural Resource Management Norway

## Abstract

Methane uptake and diversity of methanotrophic bacteria was investigated across six hydrologically connected wetlands in a mountainous forest landscape upstream of lake Langtjern, southern Norway. From floodplain through shrubs, forest and sedges to a *Sphagnum* covered site, growing season CH_4_ production was insufficiently consumed to balance release into the atmosphere. Emission increased by soil moisture ranging 0.6–6.8 mg CH_4_ m^−2^ h^−1^. Top soils of all sites consumed CH_4_ including at the lowest 78 ppmv CH_4_ supplied, thus potentially oxidizing 17–51 nmol CH_4_ g^−1^ dw h^−1^, with highest V_max_ 440 nmol g^−1^ dw h^−1^ under *Sphagnum* and lowest K_m_ 559 nM under hummocked *Carex*. Nine genera and several less understood type I and type II methanotrophs were detected by the key functional gene *pmoA* involved in methane oxidation. Microarray signal intensities from all sites revealed *Methylococcus*, the affiliated Lake Washington cluster, *Methylocaldum*, a Japanese rice cluster, *Methylosinus*, *Methylocystis* and the affiliated Peat264 cluster. Notably enriched by site was a floodplain *Methylomonas* and a *Methylocapsa*‐affiliated watershed cluster in the *Sphagnum* site. The climate sensitive water table was shown to be a strong controlling factor highlighting its link with the CH_4_ cycle in elevated wetlands.

## INTRODUCTION

**FIGURE 1:**
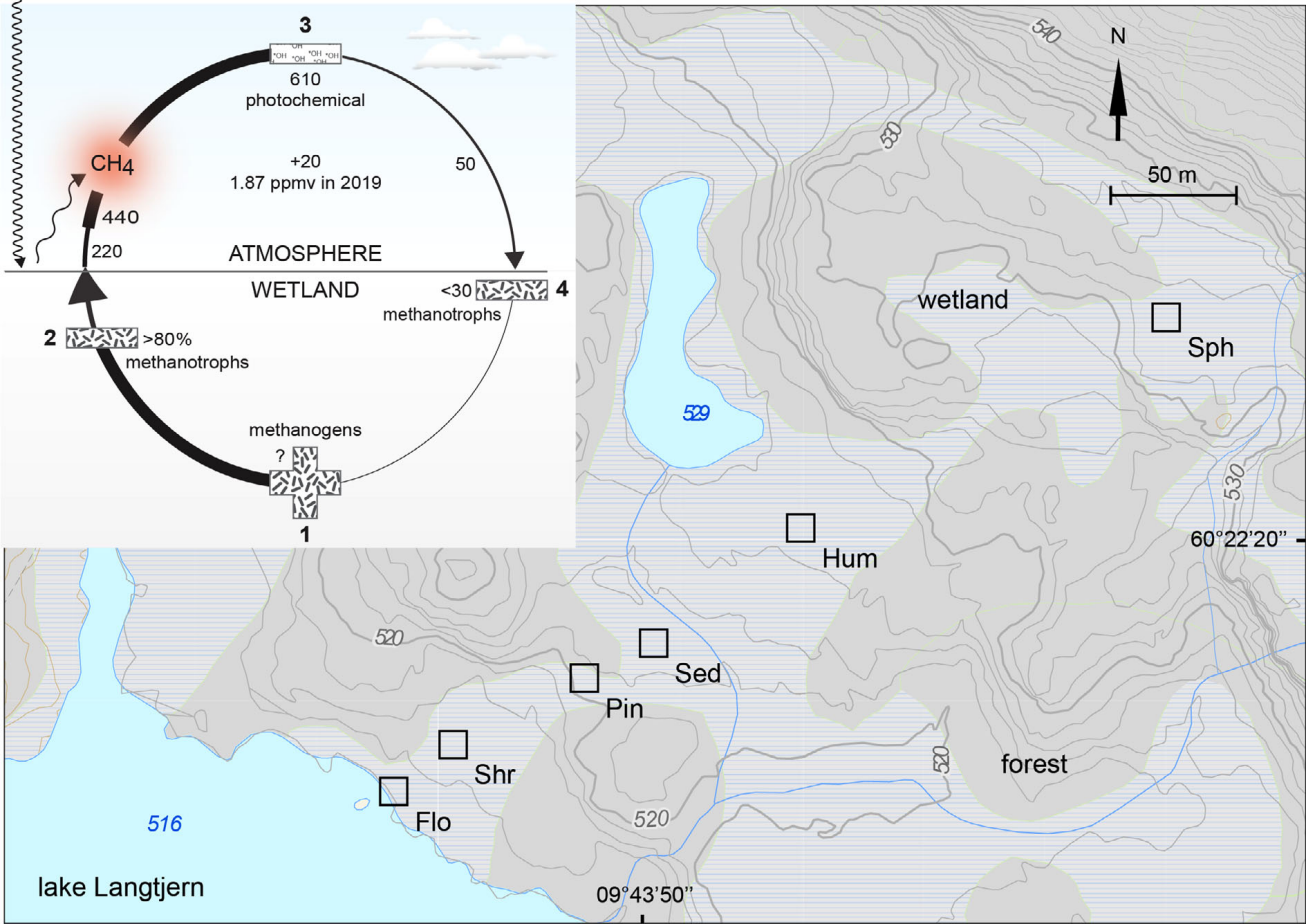
Global warming, wetland methane cycle and study sites. CH_4_ in peatlands is produced by methanogenic archaea (1), consumed by a filter of methanotrophic bacteria (2), photodegraded with hydroxyl radicals (3) and consumed from the atmosphere by methanotrophs in soils. Numbers represent millions of tonnes per year and are estimated averages, including 440 from other sources (IPCC, 2021; Reay et al., 2018). The warming result as CH_4_ reemit  infrared radiation temporarily stored as molecular bound rotational and vibrational energy (Mitchell, 1989). Owing to the accumulation of greenhouse gases over the last 100–150 years, the global temperature has increased by 1.1°C with CH_4_ contributing ~15%, during its atmospheric lifetime of ~10 years (IPCC, 2021). The study sites are seen located upstream of lake Langtjern forming a hydrologically connected gradient in the watershed (60°22′20″ N, 09°43′50″ E). Features of interest were edited onto a map downloaded from The Norwegian Water Resources and Energy Directorate (www.nve.no/english/). Flo, floodplain; Shr, shrubs; Pin, pine; Sed, sedges; Hum, hummocks and Sph, Sphagnum

Methane released into the atmosphere accumulates by ~20 Tg (million tonnes) each year trapping an increasing amount of terrestrial radiation (Figure 1). CH_4_ contributes ~15% to global warming and is the most important greenhouse gas after CO_2_ (Conrad, 2009; Dean et al., 2018) and water vapour (Mitchell, 1989). Growing evidence suggests that warming is amplified at high elevation such as in mountainous subalpine environments (Pepin et al., 2015). The warming reduces frost and snow and intensifies rain, flooding and drought, impacting local ecosystems and organisms including the major source of water for large human populations in lower elevation regions (IPCC, 2021). Realizing that CH_4_ increases in the atmosphere early made it necessary to understand the natural processes causing the source‐ and sink imbalance (Cicerone & Oremland, 1988).

Wetlands capture carbon with more than half of it stored north of 45° latitude in habitats such as boreal peatlands of mire fens and bogs (Reay et al., 2018). Wetlands are also the largest natural source of atmospheric CH_4_ (Conrad, 2009; Figure 1). Annually ~230 Tg of CH_4_ (22%) arise from wetlands (Reay et al., 2018). Their standing waters limit gas diffusion and oxygen and other electron acceptors like sulfate are typically low (Conrad, 2009). In the waterlogged peat layers organic matter degradation produces acetate, methanol, hydrogen and CO_2_ fuelling methanogenic Archaea that release CH_4_ (Cicerone & Oremland, 1988; Figure 1). Acetate is additionally provided in plant root exudates (Le Mer & Roger, 2001). Peat pore water can be supersaturated in CH_4_ (>1 vol%), which escapes by diffusion, bubbles, and through air‐filled aerenchyma spaces within plants (Dean et al., 2018; Le Mer & Roger, 2001; Reay et al., 2018). Field chamber measurements of CH_4_ in mountainous wetlands in Czechia (Urbanová et al., 2013), the Swiss Alps (Cheema et al., 2015), Alaska (Sebacher et al., 1986), the Qinghai‐Tibetan Plateau (Hirota et al., 2004) and Australia (McDaniel et al., 2021) revealed emissions of 0.3–15 mg CH_4_ m^−2^ h^−1^ similar to northern low‐land wetlands of Swedish mires (Nilsson et al., 2001) and a Finnish lake nearshore littoral zone (Siljanen et al., 2011).

Estimates suggest more than 80% of wetland CH_4_ never reaches the atmosphere (Le Mer & Roger, 2001; Figure 1). Wetlands are inhabited by a unique group of aerobic bacteria that can use CH_4_ as their sole carbon and energy source (Hanson & Hanson, 1996). The methanotrophs function as an effective filter capturing CH_4_ before it is released into the atmosphere (Galchenko et al., 1989). In periods of droughts and lowered water table CH_4_ may be sourced from the ~1.87 ppmv (~0.0002 vol%, ~3.5 nM dissolved) that diffuses into soil from the overlying atmosphere. Altogether soil methanotrophs consume up to ~30 Tg atmospheric CH_4_ yr^−1^ (Reay et al., 2018; Figure 1). Measurements on the drier mountainous soils revealed low (Sebacher et al., 1986; Urbanová et al., 2013) to slightly negative fluxes and in flask incubations a potential sink (McDaniel et al., 2021) similar in magnitude to low‐land wetlands (Siljanen et al., 2011).

The gene *pmoA* encoding for the beta subunit of the CH_4_‐oxidizing enzyme particulate methane monooxygenase (pMMO) is widely used as a functional marker for detecting methanotrophs in environmental samples (Dumont & Murrell, 2005). In the microarray it provides a fast and cost‐effective method to probe for predefined groups of *pmoA* in multiple samples (Bodrossy et al., 2003) with results comparable to pyrosequencing (Kip et al., 2011). Phylogeny with the *pmoA* reflects very well that of the 16S rRNA (Kolb et al., 2003) and the clustering of taxa (Tsuji et al., 1990) corresponds to the phenotypic groupings of Whittenbury et al. (1970) still forming the taxonomic foundation for methanotrophs in many ecosystems (Kalyuzhnaya et al., 2019). Most methanotrophs group as type I (Gammaproteobacteria), some as type II (Alphaproteobacteria) and a few as the phyla Verrucomicrobia (Op den Camp et al., 2009) and NC10 (Ettwig et al., 2010). There are many less understood taxa several only known by molecular detection. Three type II genera equipped with only the soluble methane monooxygenase lack *pmoA* (Dedysh et al., 2000; Vekeman et al., 2016; Vorobev et al., 2011). Among these, the wetland inhabitant *Methylocella* (Dedysh, 2011; Theisen & Murrell, 2005) prefer acetate over CH_4_ (Nazaries et al., 2013).

By the year 2100 wetland CH_4_ emissions are predicted to increase by 33%–60% because of increased temperature and precipitation causing less frost and longer periods of emissions changes intensified by latitude and altitude (Dean et al., 2018; Pepin et al., 2015). Altered temperature and hydrology may cause the peat to start decomposing shifting wetland functioning from sink to source of greenhouse gases (Salimi et al., 2021). Microorganisms are highly sensitive to perturbation (Cavicchioli et al., 2019), but CH_4_ oxidation at conditions adapted to by methanotrophic bacteria cannot be compensated for by other microbial groups (Ho et al., 2013).

Our aim was to investigate the CH_4_ sink strength associated with methanotrophs in a hydrologically connected gradient of northern boreal mountainous wetlands to facilitate understanding on scaling the fluxes to larger areas and predict the response of these complex wetlands to global warming. We measured CH_4_ emission, potential CH_4_ oxidation and probed for *pmoA* genes in six wetland sites of peatland mires in a west facing slope at 516–532 m altitude covering 400 m upstream the lake Langtjern (Figure 1). At this elevation of a typical southern Norway landscape, an array of taxa was found inhabiting net CH_4_ emitting peat soils.

## RESULTS AND DISCUSSION

Although numerous studies have characterized the source sink strength associated with soil methanotrophic bacteria, our study is new in combining flux rates, oxidation and taxonomy across a mountainous gradient of wetlands. In natural wetlands like Langjern, seasonally stable communities may be expected with CH_4_ emitted to the atmosphere as the remains of a flux that bypass and sustain methanotrophs.

### CH_4_ field emission

**FIGURE 2:**
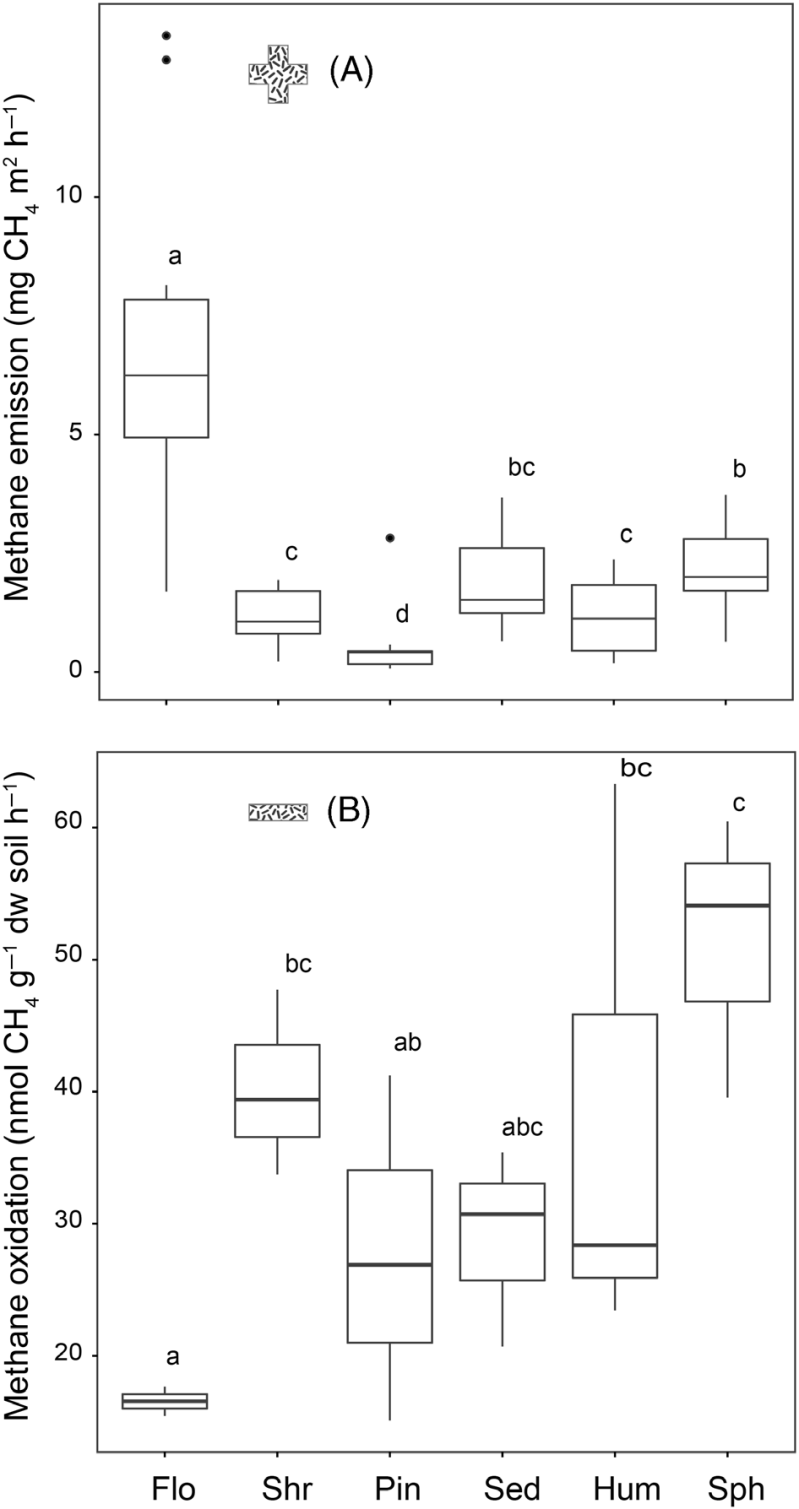
(AB) Sites methane flux rates and soils potential CH_4_ oxidation. (A) Flux rates are averaged over two 160 days growing seasons from May to October. Gas samples were collected weekly from five chambers per site, 1, 15, 30 and 45 min after chamber deployment. The rates were estimated by linear or quadratic slope changes over time (Rochette & Bertrand, 2008). (B) Potential oxidation in the sites topsoil. Equivalent to 0.5–1.5 g dw soil was dispersed in 24 ml distilled water and incubated at 15°C in triplicate 120 ml flasks continuously stirred for aeration. Methane was injected for desired initial mixing ratio (here 78 ppmv). Headspace measurements were performed using a gas chromatographic robotic system (Molstad et al., 2007). Rates were estimated by linear regression of ln transformed concentrations over 24–30 h (Jensen et al., 1998). For boxes of the plot that do not share the same letter, corresponding means are significantly different in Kruskal–Wallis multiple comparisons (*p* ≤ 0.05).

Methane fluxes were measured throughout two growing seasons using field chambers (Rochette & Bertrand, 2008) inserted into sites varying by wetland type, vegetation cover, water table, dissolved CH_4_, peat depth and pH 4.0–4.7 (Table 1; Supplementary information S1) indicating an acidic environment consistent with many peat‐accumulating wetlands. Gas chromatography (Agilent GC‐7890A; California, USA) throughout spring, summer and autumn revealed all sites released CH_4_ into the atmosphere (Figure 2A). Emission was highest from the floodplain at 6.8 ± 1.2 mg CH_4_ m^2^ h^−1^ (Figure 2A) similar to for example the Swiss Alpine fens (Cheema et al., 2015) and Swedish mires (Nilsson et al., 2001). The Flo site water table frequently raised to above the surface causing flood and a generally shallow oxic zone for CH_4_ oxidation. Methane emission is further facilitated by escape through roots and aerenchyma of plants like *Carex* and *Eriophorum* although O_2_ carried deeper by roots aid oxidation. Site Flo emitted more than twice the CH_4_ from any other site. Towards the drier sites, emissions fell with water table and pore water CH_4_ to near zero in the forest which is paludified becoming converted into  peatland (Figure 2A, Table 1). Overall, the sites growing season emission accumulated to 8.4 ± 1.2 g CH_4_ m^2^ yr^−1^.

**TABLE 1:** Overview of the wetlands vegetation and soils

Vegetation cover^a^	grasses (Flo)	shrubs (Shr)	pine (Pin)	sedges (Sed)	sedges (Hum)	sphagnum (Sph)
Peat dept (cm)	177	110	59	102	232	110
Water table (cm)^b^	0.4 (12−+30)	7.0 (25‐0)	20.3 (40‐13)	8.9 (26‐2)	11.1 (34‐3)	9.8 (24‐3)
Dissolved CH_4_ (μM)^b^	135 (30‐260)	57 (12‐160)	11 (2‐24)	38 (8‐212)	48 (11‐115)	42 (1‐162)
pH	4.1	4.3	4.2	4.1	4.0	4.7
DOC (mg L^−1^)	22.9	24.0	71.1	17.0	29.5	14.9
Total C (%)	48.4	55.2	51.0	52.5	52.5	51.7
Total N (%)	1.3	2.8	2.1	2.9	2.6	2.3
C/N	39	20	24	18	20	23
V_max_ (nmol g^−1^ dw h^−1^)^b^	185 (28)	171 (25)	116 (15)	197 (33)	188 (28)	440 (84)
K_m_ (nM)^b^	2490 (749)	722 (395)	883 (360)	772 (504)	559 (355)	1254 (657)
Shannon	1.83	2.04	2.33	2.61	2.74	1.84

^a^ Indicated dominant with largest bog being hummocked (detailed in Supplementary S1).

^b^ Below surface, brackets enclose min max values of six replicates and standard error of three replicates.

### CH_4_ oxidation potential

Cores down to 80 cm depth were collected from each site, sectioned and subsampled to flasks for gas chromatography (Supplementary Information S1). Stirred as slurries at 15°C all depths consumed CH_4_ and as expected mostly in the topsoil (data not shown). Triplicate topsoil 0–20 cm samples (Flo 0–10 cm) collected the following summer confirmed the oxidation (Figure 2B). As seen for other soils (Jensen et al., 1998) CH_4_ appeared to be consumed at a log linear scale reaching no observed threshold. Within the 24–30 h rate estimation period, the lowest initial 78 ppmv supplied fell to 1 ppmv by, for example, the hummocked *Carex* site sample. Potential oxidation was highest for the *Sphagnum* soil (51 ± 6 nmol CH_4_ g^−1^ dw h^−1^) and lowest for the Flo soil (17 ± 0.6 nmol CH_4_ g^−1^ dw h^−1^; Figure 2B). Rates compare to those observed in a sub‐alpine Australian bog (McDaniel et al., 2021) and a Finnish wetland including overall flux pattern confirming that hydrology affects methanotrophic activity and community composition (Siljanen et al., 2011). The relatively low rate observed for site Flo might reflect oxidation by methanotrophs associated with the *S. cuspidatum* lawns in the aboveground plant parts (Raghoebarsing et al., 2005) not included in the oxidation assay here. Increasing the flasks headspace initial CH_4_ concentration from 78 to about 5000 ppmv suggested highest potential CH_4_ oxidation for the Sph soil (V_max_ 440 ± 84 nmol g^−1^ dw h^−1^) and lowest half saturation (K_m_ 559 ± 355 nM) for the Hum soil (Table 1; Supplementary information S1).

### Methanotrophs abundant in all sites

**FIGURE 3:**
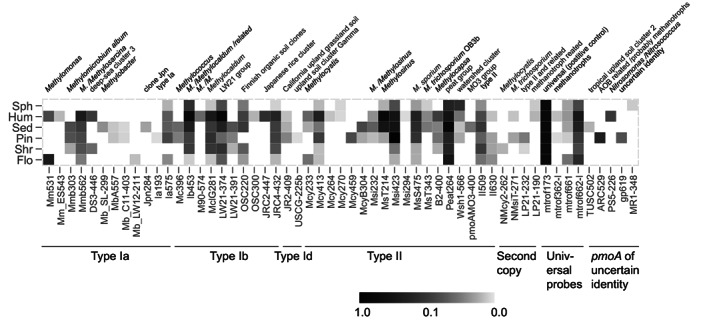
Abundance of soil methanotrophic taxa assessed using the *pmoA* microarray of Bodrossy et al. (2003). Every new taxon is named adopting mostly Knief (2015). The probes target species, groups of species and genera as well as higher taxonomic groups of methanotrophs and related bacteria. Numbers at the end of the probe names refer to the relative positions on the *pmoA* gene of *Methylococcus capsulatus* (Bath). A value of 1.0 indicates maximum achievable signal for an individual probe, a value of 0.1 indicates that about 10% of the total PCR product hybridized to that probe (Stralis‐Pavese et al., 2004). Values are normalized to the positive control probe mtrof173. To enhance identification blastn was used with the Entrez query ‘pmoA Methylo* not [uncultured]’. This version of the array cover most of the diversity among type I and type II methanotrophs except *Methylocella*, *Methyloferula*, *Methyloceanibacter*, *Crenothrix*, and NC10 *Methylomirabilis* and Verrucomicrobia (Abell et al., 2009; Siljanen et al., 2011).

DNA was extracted from ~0.3 g topsoil using the FastDNA spin kit for soil with method of Pan et al. (2010) and *pmoA* sequences amplified in two rounds using primers A189/A682 and A189/mb661 for increased yield and specificity (Siljanen et al., 2011; Supplementary information S1). The recovered sequence types were displayed as a semi‐quantitative profile using a *pmoA* microarray (Bodrossy et al., 2003). Of the 135 applied probes, 55 were positive (Figure 3). A striking observation was the many poorly understood methanotrophs detected. Among signals affiliated with *pmoA* from type I methanotrophs were *Methylococcus* (probe ib453) and *Methylocaldum* (probe MclG281) from hot springs, a Japanese rice cluster *Methylogaea* (probe JRC4‐432) from paddy soil (Knief, 2015) and especially an until recently uncultured organic soil cluster OSC more broadly defined as freshwater cluster LW21, relatively abundant in samples from every site (Figure 3). These are all of type Ib (formerly type X), a poorly characterized group requiring more attention (Deng et al., 2013). The most abundant LW21, was first detected in Lake Washington sediment (Auman et al., 2000) and has been found in, for example, Lake Constance sediment (Pester et al., 2004), in soils of the Finnish wetland (Siljanen et al., 2011) and in the Swiss alpine fen soils where T‐RFLP of *pmoA* transcript sequences indicated activity (Cheema et al., 2015). LW21 predominate bogs and some typical well‐drained and aerobic so‐called upland soils (Knief, 2015). A *Methylococcus*‐affiliated but spiral and microaerophilic LW21 morphotype ‘*Candidatus* Methylospira mobilis’ was enriched (Danilova et al., 2016) and isolated (Oshkin et al., 2019) from a *Sphagnum* dominated peat bog in the Tver region of north‐western Russia. Its helical cell shape and up to 6.0 μm s^−1^ velocities suggests adaptation to move through relatively viscous, partially decomposed plant debris and biofilm, to become enriched in the high methane low oxygen interphase (Danilova et al., 2016). Whether LW21 includes the novel type I methanotrophs found to be transcriptionally active in peat soil of the Moor House Nature Reserve in northern England (Chen et al., 2008) remains unclear. Clearer it seems that the role of type I methanotrophs in peatlands have been underestimated.

Likewise, apparently little influenced by environmental variation, well‐characterized type II methanotrophs co‐occurred with a poorly understood lineage of *pmoA* sequences (Figure 3). This Peat264 cluster dominated signals from such as *Methylosinus* (probes Msi423 and MsS475) and *Methylocystis* (probe Mcy413). The ubiquity of type II methanotrophs in the Langtjern samples was supported by the general probe II509 signal. Detected in a Moor House blanket bog peat (McDonald & Murrell, 1997) and since detected there (Chen et al., 2008), associated with *Sphagnum* mosses from a Dutch peat bog (Kip et al., 2011) and, for example, in the Finnish wetland soil (Siljanen et al., 2011) including oak forest soil (Radajewski et al., 2002), Peat264 has been suggested a cluster of widespread persistence in peatlands. No cultured representative or metagenome has been reported leaving the Peat264 physiology poorly understood. Using blastn with the probe sequence, its 23 nucleotides exactly matched more than 100 *pmoA* sequences including PCR amplicons from Moor House peat core sections and enrichment cultures (McDonald & Murrell, 1997). The closest cultured isolates matched 18/19 nucleotide positions and belonged to *Methylocystis* of species including *M. bryphila* H2s from the Teufelssee peat‐bog lake in north‐eastern Germany (Belova et al., 2013). *M. bryophila* H2s prefers CH_4_ and methanol and in the absence of one‐carbon compounds grows slowly on acetate (Belova et al., 2013). *Methylocystis*/*Methylosinus* dominated the microarray and clone library gene and transcript sequences of both *pmoA* and 16S rRNA at Moor House, suggesting type II to be largely responsible for the CH_4_ oxidation *in situ* (Chen et al., 2008) possibly indicating a distinction compared to the less *pmoA* type II transcript activity detected in mountainous soil (Cheema et al., 2015). *Methylocystis* are among the most abundant and metabolically active methanotrophs in northern wetlands (Dedysh, 2011; McDonald & Murrell, 1997).

### Methanotrophs enriched by site

**FIGURE 4:**
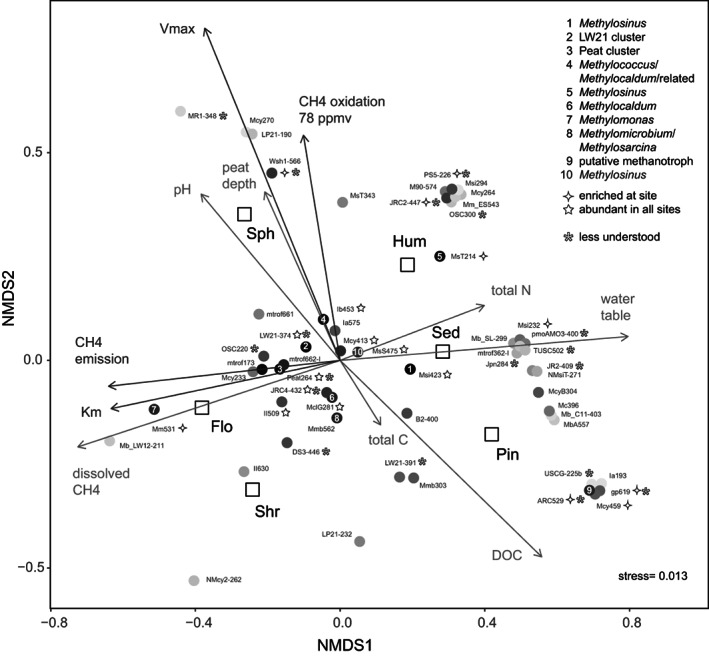
Non‐metric multidimensional scaling (NMDS) using probe signal to display methanotrophic community structure across sites. Sites are shown as six open squares. Taxa are shown as shaded dots representing all 55 positive probes. The shading indicates probe signal intensity reflecting taxon abundance. Environmental variables, emission, oxidation, K_m_ and V_max_ was added onto the two‐dimensional Bray‐Curtis distance representation of the communities using envfit. Arrow directions indicate increasing correlation of taxa with that variable. Strongest correlation was found with water table distance below surface (*r* ^2^ = 0.63, *p* = 0.17).

The probe signals were clustered to better understand the environmental variation. Linear ordination was used as detrended analysis indicated a homogeneous dataset of relatively low standard deviation. As shown by nonmetric multidimensional scaling (Figure 4), taxa (dots) scattered such that most of the variation (first axis) separated communities of Flo, Shr and Sph from Pin, Sed and Hum (squares). Analysis of variance (ANOVA) indicated significantly different species composition patterns between the two groups (*F*_(1,328)_ = 8.807, *p* = 0.003). Similarity percentages analysis (SIMPER) demonstrated communities of the former three separated from the latter owing to a higher abundance of especially *Methylomonas* (probe Mm531), water shed cluster (probe Wsh1‐566) and a lower abundance of several taxa the most contributing to the dissimilarities being *Methylosinus* and Peat264. This seems to agree with the hypothesis of type I being more active at high CH_4_ concentration (copiotrophic) while type II fit more within the stress tolerator (oligotrophic) categories (Ho et al., 2013). Adding environmental variables (Figure 4), the strongest community structure relationship was observed for the water table which together with pore water CH_4_, emission, and total N was driving differences between the two groups. Within group differences were driven by peat depth, pH and dissolved organic carbon. Influence on physiology was indicated by including K_m_, V_max_ and CH_4_ emission (Figure 4).

*Methylomonas* has been shown to assimilate CH_4_ into phospholipid fatty acids in a Dutch floodplain soil (Bodelier et al., 2013) and is probably also oxidizing CH_4_ at Langtjern. Also using ^13^CH_4_, 10%–15% of the carbon consumed by peat soil accumulated in a *Sphagnum* specific sitosterol (Raghoebarsing et al., 2005). *Methylocapsa* or *Methylocella* (93% 16S rRNA gene identity) was suggested to be involved in both the efficient recycling of CH_4_ and the high organic carbon burial. However, no *pmoA* or *mmoX* was recovered and the status as a methanotrophic symbiont appears unclear (Dedysh, 2011). Nonetheless, *Sphagnum* is an important peat‐forming plant abundant or interspersed in all sites at Langtjern. The Sph sample was enriched in a poorly understood water shed cluster intriguingly also affiliated with *Methylocapsa*. Moreover, an upland soil cluster USCα, that is, RA14 (Holmes et al., 1999) genome draft revealed a widespread *Methylocapsa*‐like methanotroph (Pratscher et al., 2018) growing on atmospheric CH_4_ possibly supported by H_2_ and CO as well as polyhydroxybutyrate (Tveit et al., 2019). In blastn, the probe Wsh1‐566 sequence did not produce exact matches with cultured representatives but matched sequences from a US bottom land watershed (Ogram et al., 2006) and by one mismatch to sequences of a Moor House peat cluster (Chen et al., 2008). The MHP cluster has been more frequently detected in soils with higher CH_4_ supply such as peatlands and wetlands than in upland soils (Knief, 2015). MHP form a sister clade with a California Jasper Ridge soil clade (Horz et al., 2005) and the USCα (Knief, 2015), none of which were detected at Langtjern.

Similar to such as Finnish fen habitats (Peltoniemi et al., 2016), a higher diversity of methanotrophs associated with the drier sites (Table 1, Figure 4). These taxa included *Methylomicrobium*/*Methylosarcina*, *Methylobacter*, another Japanese Rice cluster, type Id methanotrophs, *Methylocystis*, *Methylosinus* and *Methylocapsa*. The Shannon diversity of these samples (Table 1) approached the Finnish wetland soil (Siljanen et al., 2011). Drainage from a slope and sand/rock may enrich for type II methanotrophs in wetland soils (McDaniel et al., 2021). At Langtjern, the forest site was relatively steep, and the hummocks raised above the surrounding site. The Pin‐site had the lowest water table, relatively low CH_4_ affinity and the strongest microarray signal (*Methylosinus* probe Msi423). In pairwise comparisons site Hum revealed the most dissimilar methanotrophic community (Tukey HSD *p* < 0.085). Type II methanotrophs fix N_2_ (Hanson & Hanson, 1996) and the Hum and Pin soils were enriched in organic matter and nitrogen (Table 1, Figure 4). Their ammonium oxidizers (probe PS5‐226) and putative methanotrophs (probe ARC529) are strongly site enriched signals (Figure 3).

### CH_4_ oxidation and methanotroph identity

Our study is limited to aerobic methanotrophs and previously sequenced *pmoA* genes. Relevant but undetected is the potential for anaerobic CH_4_ oxidation. Its rate was only included in fluxes from the field as O_2_ levels in the flasks never dropped below 16% vol/vol. In the boggy Australian site, 16S rRNA gene sequences affiliated with the nitrite‐reducing *Methylomirabilis* (mismatching *pmoA*) that dwarfed those of type I and II methanotrophs (McDaniel et al., 2021). The other methanotrophs that would be undetected are *Methylocella* (Dedysh et al., 2000) and *Methyloferula* (Vorobev et al., 2011) as they lack *pmoA* but are present in wetland soils (Dedysh, 2011; Theisen & Murrell, 2005). It is unclear how much CH_4_ is consumed by *Methylocella* as they may use acetate (Dedysh, 2011) and less CH_4_ oxidation by these methanotrophs was suggested at Moor House where *pmoA* transcripts were more abundant than *mmoX* transcripts (Chen et al., 2008). In Dutch *Sphagnum* moss and Qinghai‐Tibetan peatlands, the *mmoX* gene sequences were also found low (Deng et al., 2013; Kip et al., 2011).

The soil CH_4_ sink should be considered a net effect of CH_4_ consumption and production processes, which may occur either simultaneously or separated in time and space (Dunfield, 2007). Given that water table exerts major control on wetland CH_4_ fluxes by stimulating methanogenesis and inhibiting methanotrophy (Le Mer & Roger, 2001; Nazaries et al., 2013) survival based on CH_4_ as the sole carbon and energy source may risk starvation. Atmospheric CH_4_ consumption was early reported for peat soil, through a drought period in the Great Dismal Swamp wetland, Virginia USA (Harriss et al., 1982). At Langtjern, oxidation of atmospheric CH_4_ by type Id, USCγ (Knief et al., 2003), JR2 (Knief, 2015) or any second *pmoA* copy detected seems low because of their faint signals at strength of 2.9%–1.0% of the reference value for positive detection, which is close to the detection limit of the array (5%). Instead, the more abundant type II, Wsh1‐566 and Peat264 methanotrophs might be involved. Recently, transcriptomics has revealed species of conventional *Methylosinus* and *Methylocystis* as well as type I methanotrophs such as *Methylosarcina* to consume CH_4_ with ‘high affinity’ for 2 weeks (Cai et al., 2016) following the previously used incubation of paddy soil at elevated CH_4_ concentration (Bender & Conrad, 1992) to mimick the *in situ* flush feeding expected from fluctuations in the soil water table. Evidence is growing for the facultative lifestyle and niche flexibility of methanotrophs (Bay et al., 2021).

## CONCLUSIONS

The wetlands at Langtjern suggest Norwegian mountainous landscapes interspersed with wetlands are a considerable source of CH_4_ into the atmosphere. However, the scale of this source is unclear, especially across the whole boreal region, as the output of the CH_4_ cycle vary by the complexity of the terrain and conditions such as moisture, plant cover, nutrients and other factors regulating peat soil organismal activity. The Langtjern methanotrophs structured into communities apparently reflecting adaptation. Abundance was indicated higher of type I in wetter high‐CH_4_ sites and higher of type II in drier low‐CH_4_ sites more likely with a potential for sustained atmospheric oxidation. Which methanotrophs primarily drive the boreal mountainous wetlands sink strength is unclear. Specifically, whether indicator methanotrophs or distinct methanotrophic communities exist in these environments is unknown. For example, representative host‐associations could exist beyond *Sphagnum*. As climate change progresses, higher altitude soils may destabilize faster, providing foresight into future changes of corresponding soils at lower altitude (Pepin et al., 2015). Since microorganisms respond quickly to environmental change (Cavicchioli et al., 2019) and poorly characterized taxa are less predictable, slight changes in surface peat hydrology can have large consequences for the CH_4_ flux.

## CONFLICT OF INTEREST

We have no conflict of interest.

## Supporting information


**Appendix S1:** Supporting Information
