# Supplementary material for: Activity and abundance of methanotrophic bacteria in a northern mountainous gradient of wetlands
Source: Environ Microbiol Rep. 2023 Feb 14;15(3):206–15. doi: 10.1111/1758-2229.13137 (PMC10464705; doi:10.1111/1758-2229.13137)
Supplement: Supplementary file 1 — Appendix S1: Supporting Information [file EMI4-15-206-s001.docx]

**Supporting Information**

Additional Supporting Information may be found in the online

version of this article at the publisher’s web-site:

*Study sites, sampling and metadata*

In Norway, wetlands cover an estimated area of 8.9% (28,777 km^2^) including 4.0% peatland forests while Alpine mountain heaths cover 24% of the land (Bryn *et al*., 2018). Our study sites are wetlands at 516 to 532 m altitude upstream of lake Langtjern in southern Norway (Fig. 1; 60º 22' 20'' N, 09º 43' 50'' E). Lake Langtjern has been a research station for national monitoring programs on acid rain since 1972 and since 2010 including a weather station (de Wit *et al*., 2018). The Langtjern area is dominated by 80% mature low- to unproductive Scots pine (*Pinus sylvestris*) forest and patches of Norway spruce (*Picea abies*) interspersed with 20 % peatland, in a geology of till from felsic gneisses and granites, with thin mineral soils in exposed positions and deeper peaty soils in depressions and close to streams (de Wit *et al*., 2018). Peatlands are mostly acidic bogs with some contact to groundwater. While the bogs are usually treeless, some of the forest soils are paludified, i.e. have a large share of sphagnum mosses in their ground vegetation. Climate is reported for the years 1986 to 2015 and 2015 to 2019 with average temperatures 2.5 and 4.1 °C and average precipitation 901 and 921 mm, respectively (de Wit *et al*., 2018; Clayer *et al*., 2021). Seasonally, mean monthly highest and lowest temperatures are around 20 and -10 °C, precipitation is highest in autumn and snow depths vary around 100 cm (https://aquamonitor.niva.no/langtjern/). Langtjern represent a typical southern Norway landscape at this elevation. The complexity of such landscapes poses a challenge to scaling the CH_4_ source strength and methanotrophic abundance to greater areas.

The sites are in the following briefly described based on own observations, a vegetation map (Hofsten, 1975) and land cover vegetation types (Bryn *et al*., 2018). All sites are wetlands accumulating peat considered falling into the terminology of peatland mires and mostly bogs (Wheeler and Proctor, 2000). The sites form a hydrological network much influenced by topographically controlled surface throughflow. The floodplain site (Flo) is a riparian zone frequently inundated and mostly vegetated by *Sphagnum cuspidatum* moss, some *Carex* sedges of various unidentified species, *Eriophorum vaginatum* cotton grass and other grasses. The shrubs site (Shr) was never found inundated and is a bog dominated by *Sphagnum* mosses, *Empetrum nigrum* crowberry *Calluna vulgaris* heather and bush-like *Betuala* birch. The forest site (Pin) is paludified i.e. in the process of being converted to peatland and the *Pinus sylvestris* Scots pine has an undergrowth of *Betula*, *Vaccinium myrtillus* blueberry and *V. uliginosum* bog blueberry. The sedge site (Sed) is a bog dominated by various *Carex* species including a large portion of *E. vaginatum*. The hummock site (Hum) is a bog dominated by *Carex* including *E. vaginatum* and *C. vulgaris* but with marked hummocks. The Sphagnum site (Sph) is a bog with a lawn of *Sphagnum* mosses without hummocks.

Sampling of peat depth, other metadata and the field fluxes, was performed during the growing seasons of 2006 and 2007. The peat depth was measured manually by means of a narrow rod probe. Depth of the water table was measured manually in piezometers (custom made from plastic tubing) permanently installed close to the gas measurement plots. Dissolved soil pore water gases including CH_4_ were recovered from 9 cm MacroRhizons (Rhizosphere Research Products, NL) installed permanently at different depths. Samples were taken slowly into 20 ml disposable syringes equipped with 2-way valves. The mixture of water and gas obtained was injected into evacuated 10 ml vials, which were equilibrated to 1 atmosphere by carefully adding He before headspace analyses by the gas chromatograph. Dissolved CH_4_ concentrations at *in situ* temperature were back calculated using the Henry constant. Using a box corer (4 x 6 cm), soils were collected from the sites. The samples green parts i.e. living plants were removed before analyses. Soil pH was measured in a 1:2.5 slurry of distilled water using a pH meter (Thermo Orion Star A211). Dissolved organic carbon was measured using a Shimadzu TOC-V CPN analyser (Bruker, Germany) equipped with a non-dispersive infrared (NDIR) detector. Total C and N were analysed by Dumas combustion on a LECO CNS analyser (Leco CHN628). At each site, five PVC collars (30 cm diameter, 50 cm high) were permanently installed to a depth of 40 cm at a distance of approximately 1 m from each other. The collars served as bases for static, vented PVC chambers (30 cm diameter, 35 cm high) operated from boardwalks to avoid ebullition (Rochette and Bertrand, 2008). The chambers were sampled weekly for 160 days from May 3 to October 10 of in both years. Using a syringe, 4 gas samples were collected 1, 15, 30 and 45 min aften chamber deployment and immediately transferred to 10 ml pre-evacuated glass vials crimp sealed with butyl rubber septa (Chromacol), awaiting headspace analyses by the gas chromatograph. Thereafter temperature inside the chamber was measured by a handhold digital thermometer.

In September 2010 soil cores were collected and sectioned to measure potential CH_4_ oxidation and extract DNA. Samples to be extracted for DNA were placed in 50 ml Nunc tubes and shipped on dry ice to the University of Bergen. Sampling was repeated in September 2011 to collect topsoil 0-20 cm samples (Flo 0-10 cm) in triplicates for potential CH_4_ oxidation and kinetic experiments. For the potential CH_4_ oxidation, 0.5 - 1.5 g wet weight peat soil was suspended in 24 ml distilled water in 120 ml serum flasks closed with crimp sealed butyl rubber septa (Chromacol) and spiked with CH_4_. The flasks were equipped with magnetic stirrers and placed on submersible stirring plates in a temperature controlled water bath adjusted to 15ºC. The water bath is placed under the robotic arm of an autosampler (GC-PAL, CTC, Switzerland). Everly approximately 3-4 hours, a sample of ~1 ml was automatically transferred to the gas chromatograph equipped with an FID (Agilent GC-7890A) using a peristaltic pump. To keep the pressure in the flask at ~1 atm, helium (plus residual sample) was automatically pumped back into the flasks. The resulting dilution was evaluated in dry flasks filled with certified standards. Corrections for dilution and dissolution of CH_4_ in water were carried out as described by Molstad *et al*. (2007).

*Kinetic experiments*

Measurements were transformed to mol taking into account the molecular weight of CH_4_, the headspace volume, the liquid volume, and the solubility of CH_4_ in water (Yamamoto *et al*., 1976). Potential CH_4_ oxidation rates were calclulated by linear regression of ln-transformed nmol CH_4_ concentration decline over the first 24 to 30 hours (r^2^ linearity >98%) to estimate first order rate constants (C= C_0_ • e^-kt^) as before (Jensen *et al*., 1998). The apparent CH_4_ half saturation constant (K_m_) and the capacity for CH_4_ oxidation (V_max_) was calculated from Eadie-Hofstee plots based on rates and average CH_4_ concentration per time increment (Bender and Conrad, 1992). The reaction rate (velocity; v) was raised using seven initial CH_4_ mixing ratios (substrate concentration; [S]). K_m_ was determined by the slope and V_max_ by the y-axis intercept of the regression line v= K_m_ • v/[S] + V_max_ (i.e. y= ax + b) in v over v/[S] (Fig. S1a). Outliers indicated using outlierTest were removed from samples. Shr (1 outlier), Sed (3 outliers), Hum (3 outliers). By inserting values for K_m_, V_max_ and S into the equation v = ([S] • V_max_)/([S] + K_m_) Michaelis-Menten kinetics were simulated (Fig. S1b). At S sufficiently low to be ignored the specific affinity V_max_/K_m_ indicates how fast limiting substrate is metabolized and can be taken as a measure of oligotrophy (Dunfield, 2007). While V_max_ reflects the number of active methanotrophs K_m_ reflects their combined enzyme efficiency. K_m_ is an intrinsic physiological trait of the soil methanotrophic community.

*DNA extraction and microarray analysis*

To extract DNA, 0.3 g of freshly thawed top soil samples from 0-20 cm depth (Flo 0-10 cm) was shaken horizontally (100 rpm min^-1^) with 780 µl lysis buffer (5 mg lysozyme ml^-1^) in a multimix FastPrep tube for 30 min at 37 ˚C. MT buffer (122 µl) was added, and the shaking continued in the FastPrep instrument (30 s at 5.5 m s^-1^). Samples were centrifugated (15 min 10,000 rpm) and 700 µl supernatant collected. The pellet was re-extracted by re-shaking with 500 µl lysis buffer and 50 µl MT buffer. Both supernatants (700 µl each) were in separate Eppendorf tubes each added 5 µl proteinase K (10 mg ml^-1^) and incubated at 65 ˚C for 30 min. Proteins were removed by phenol:chloroform:isoamyl alcohol (25:24:1) extraction followed by chlorform:isoamyl alcohol (24:1) extraction. Remaining proteins were precipitated from pooled extracts (2 x 700 µl) in 125 µl potassium acetate (7.5 M) during incubation on ice (5 min) before pelleted by centrifugation (10 min 10,000 rpm). The supernatants were each added 700 µl binding matrix and mixed for 5 min on a rotator. Following centrifugation (1 min 10,000 rpm) the pellet was resuspended in wash buffer (500 µl). The suspension was added into a Spin filter and centrifuged (1 min 10,000 rpm). The pellet was washed again and centrifuged to dry (10 sec 10,000 rpm). Elution was performed twice with 50 µl TE (pH 8.0). Assessment of the DNA was performed following agarose gel electrophoresis and ethidium bromide staining of a 5 µl aliquot (Fig. S2). The DNA was lyophilized and sent to the University of Eastern Finland for *pmoA* microarray analysis.

PCR reaction mix for the microarray analysis contained 2 x Premix F (Epicentre), 25 pmol each primer, 1 U Taq polymerase (Invitrogen) and 50 ng DNA in triplicates and these triplicates were pooled together. Step one uses primers A189 (5′-GGNGACTGGGACTTCTGG-3′) + A682-T7 (5′-TAATACGACTCACTATAGGAASGCNGAGAAGAASGC- 3′) (Holmes *et al*., 1995). Step two is more specific towards *pmoA* excluding *amoA* of the related ammonia monooxygenase enzyme and further amplifying the product by A189 + mb661-T7 (5′ -TAATACGACTCACTATAGCCGGMGCAACGTCYTTACC-3′) (Costello and Lidstrom, 1999). Target labelling, hybridization and scanning were performed as described previously with triplicated hybridizations of probes per sample on the array (Stralis-Pavese *et al*., 2004).

*Data analysis*

All statistical analyses were performed in R (R Core Team, 2020) using vegan, tidyverse, scales, agricolae and car (packages). Data were tested for normal distribution (shapiro) and homogeneity of variance (var) with difference between means tested accordingly. Graphs were created in R using ggplot2 and illustrations were created in Adobe Illustrator.

**

**

**Supplementary Fig. S1ab.** Calculation of apparent CH_4_ oxidation capacity V_max_ and apparent CH_4_ half saturation constant K_m_. **a,** Eadie-Hofstee plot indicating use of the oxidation kinetics regression line to calculate V_max_ (intersect) and K_m_ (slope) for site Flo. **b,** the corresponding Michaelis-Menten plot.

**
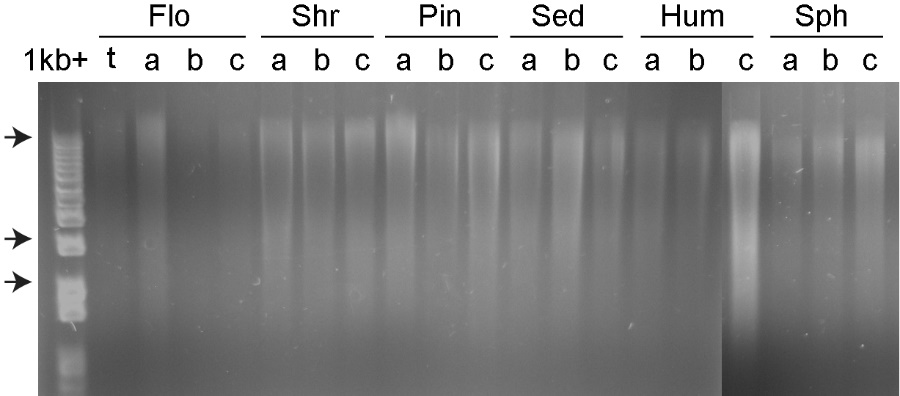
**

**Supplementary Fig. S2.** Assessment of the soil samples extracted DNA (5 µl per lane). Depth horizon is labelled for each site; t, top green parts; a, 0-20 cm (Flo 0-10 cm); b, 20-40 cm; c, 40-60 cm. Arrows point at 12,000, 2,000 and 1,000 bp band of marker 1 kb Plus loaded by 200 ng into this 1% agarose gel.

**Additional references**

Bryn, A., Strand G.‐H., Angeloff M., and Rekdal Y. (2018) Land cover in Norway based on an area frame survey of vegetation types. Norsk Geografisk Tidsskrift‐Norwegian Journal of Geography. **72**:1-15.

Clayer, F., Thrane, J.-E., Brandt, U., Dörsch, P., and de Wit H.A. (2021) Boreal headwater catchment as hot spot of carbon processing from headwater to fjord. JGR Biogeociences **126**: e2021JG006359.

Costello, A.M., and Lidstrom, M.E. (1999) Molecular characterization of functional and phylogenetic genes from natural populations of methanotrophs in lake sediments. Appl Environ Microbiol **65**: 5066-5074.

Hofsten, J. (1975) Vegetation map Langtjern, project SNSF Acid rain impact on forest and fish (In Norwegian). Jorddirektoratet avdeling for jordregister og NISK (now Norwegian Institute of Bioeconomy Research).

Holmes, A.J., Costello, A., Lidstrom, M.E., and Murrell, J.C. (1995) Evidence that particulate methane monooxygenase and ammonia monooxygenase may be evolutionarily related. FEMS Microbiol Lett **132**: 203-208.

R Core Team. (2020) R: A language and environment for statistical computing. R Foundation for Statistical Computing, Vienna, Austria. URL https://www.R-project.org/.

de Wit, H.A., Couture, R.-M., Jackson-Blake, L., Futter, M.N., Valinia, S., Austnes, K., *et al*. (2018) Pipes or chimneys? For carbon cycling in small boreal lakes, precipitation matters most. Limnol Oceanogr Lett **3**: 275-284.

Wheeler, B.D., and Proctor, M.C.F. (2000) Ecological gradients, subdivisions and terminology of north-west European mires. J. Ecol. **88**:187-203.

Yamamoto, S., Alcauskas J. B., and Crozier T. E. (1976) Solubility of methane in distilled water and seawater. J Chem Eng Data **21**:78-80.
